# BRCA1 regulation on β-hCG: a mechanism for tumorigenicity in BRCA1 defective breast cancer

**DOI:** 10.1038/oncsis.2017.75

**Published:** 2017-09-04

**Authors:** S K Sengodan, R Nadhan, R S Nair, S K Hemalatha, V Somasundaram, R R Sushama, A Rajan, N R Latha, G R Varghese, R k Thankappan, J M Kumar, A Chil, T V Anilkumar, P Srinivas

**Affiliations:** 1 Cancer Research Program, Rajiv Gandhi Centre for Biotechnology, Thiruvananthapuram, Kerala, India; 2 Animal House, Centre for Cellular and Molecular Biology, Hyderabad, India; 3 Department of Gynecologic Oncology, Kielce Oncology Center, Kielce, Poland; 4 Experimental Pathology, Sree Chitra Tirunal Institute for Medical Science and Technology, Thiruvananthapuram, Kerala, India

## Abstract

Human chorionic gonadotropin β (β-hCG) has been implicated in breast tumorigenesis. However, the role of this hormone is highly controversial as certain studies suggest it has anti-tumor properties while others have found it to be pro-tumorigenic. To unveil the truth, we have analyzed the expression of β-hCG in breast cancer. We identified for the first time that β-hCG expression is linked to BRCA1 status and its overexpression is seen in BRCA1 mutated breast cancer cells, BRCA1 conditional knockout mouse breast cancer tissues and BRCA1 floxed basal cell carcinoma (BCC) tissues. An analysis of three large, transcriptomic data sets from TCGA (The Cancer Genome Atlas) expression profile confirmed the inverse correlation between BRCA1 and β-hCG in human breast cancer. Using ChIP and luciferase assays, we also demonstrated that the cancer cells with wild-type but not mutant BRCA1 directly repress the expression of β-hCG by binding to its promoter. Further, β-hCG promotes migration and invasion predominantly in BRCA1 mutant breast cancer cells. Interestingly, stable overexpression of β-hCG in BRCA1 mutant but not wild-type breast cancer cells results in the formation of spheres even on monolayer cultures. The cells of these spheres show high expression of both EMT and stem cell markers. Since β-hCG belongs to a cysteine knot family of proteins like TGFβ and TGFβ signaling is deregulated in BRCA1 defective tumors, we checked whether β-hCG can mediate signaling through TGFβRII in BRCA1 mutated cells. We found for the first time that β-hCG can bind and phosphorylate TGFβRII, irrespective of LHCGR status and induce proliferation in BRCA1 defective cells. Our results confirmed that there exists a transcriptional regulation of BRCA1 on β-hCG and BRCA1 mutation promotes β-hCG mediated tumorigenesis through TGFβRII signaling. Thus inhibiting β-hCG-TGFβRII could prove an effective treatment strategy for BRCA1 mutated tumors.

## Introduction

Human chorionic gonadotropin (hCG), a heterodimeric molecule with α and β subunits, is mainly secreted during pregnancy by the trophoblastic cells to promote implantation of the embryo. Many studies however, have revealed that the β-subunit of hCG (β-hCG) has an independent function and is frequently associated with non-trophoblastic malignant tumors, mainly in ovarian, prostate and breast tissues.^1, 2^ However, its exact role in tumorigenesis is still unclear. Reports suggest that β-hCG can inhibit apoptosis^3^ or stimulate the growth of cancer cells and that elevated serum levels of β-hCG correlate with increased aggressiveness of the cancer.^4, 5^ Further, it has been shown that β-hCG promotes the invasion of prostate cancer cells by activating ERK1/2 and MMP-2, as well as decreasing the expression of E-cadherin in prostate cancer cells thus leading to poor disease prognosis.^6, 7, 8^ Interestingly, anti β-hCG vaccines have been developed and found to be active against β-hCG expressing cancer cells *in vitro*.^9, 10^ On the contrary, it has also been found that β-hCG can induce apoptosis in breast cancer.^11, 12^ Hence, there exists a currently unknown variable that contextually determines the effects of β-hCG in breast cancer.

BRCA1 mutation predisposes to tumors mainly to the breast and ovaries, though the exact reason for the said tissue specificity still remains a mystery. The role of hormonal factors, specifically estrogen/estrogen receptor α (ER-α) had been hypothesized as the major contributing factor for this, as BRCA1 has ligand-dependent and independent transcriptional control over estrogen receptor.^13, 14^ However, tumor progression in BRCA1 defective condition could not be controlled by inhibiting estrogen receptors as majority of BRCA1 defective cancers are ER-α negative.^15, 16, 17^ In addition to estrogen and progesterone, hCG is known to be a critical factor for the development and differentiation of the breast tissue. Therefore, we hypothesize that the other possible reason for the development of BRCA1 defective triple negative breast cancer could be the influence of β-hCG, which has not been analyzed till now. Here, we demonstrate that β-hCG can promote tumor progression by inducing TGFβRII, specifically and selectively in BRCA1 defective breast cancer cells.

## Results

### BRCA1 regulates the expression of β-hCG

**Figure 1:**
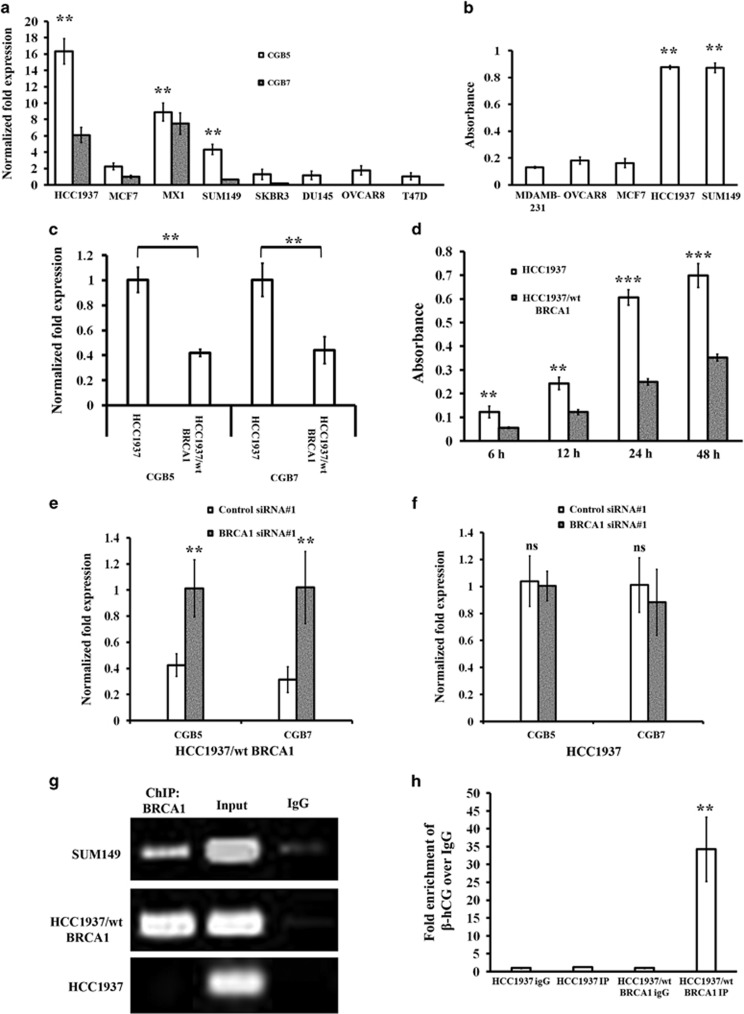
Expression and regulation of β-hCG by BRCA1 in breast cancer. All the experiments were done in triplicates. All error bars in the graphs represent s.d. (**a**) qRT-PCR of β-hCG expression in breast/ovary/prostate cancer cell lines. (**b**) β-hCG expression in cancer cell lines using ELISA. (**c**) qRT-PCR analysis of CGB5 and CGB7 expression in HCC1937 and HCC1937/wt BRCA1. Expression was normalized to HCC1937 and HCC1937/wt BRCA1, respectively. (**d**) β-hCG protein expression at different time points in HCC1937 and HCC1937/wt BRCA1 using ELISA. Expression of CGB5 and CGB7 in BRCA1 silenced (**e**) HCC1937 and (**f**) HCC1937/wt BRCA1 by qRT-PCR. Expression was normalized to scrambled siRNA transfected HCC1937 and HCC1937/wt BRCA1 (**g**) Semi-quantitative RT-PCR amplification of CGB5 promoter immunoprecipitated with anti-BRCA1 antibody using fragmented chromatins from HCC1937, SUM149 and HCC1937/wt BRCA1. Normal chromatin fragments were used as input. Chromatin samples immunoprecipitated with IgG was used as negative control. (**h**) qRT-PCR amplification of CGB5 promoter immunoprecipitated with anti-BRCA1 antibody and IgG in HCC1937 and HCC1937/wt BRCA1. ***P<0.001; **P<0.01; *P<0.05.

**Figure 2:**
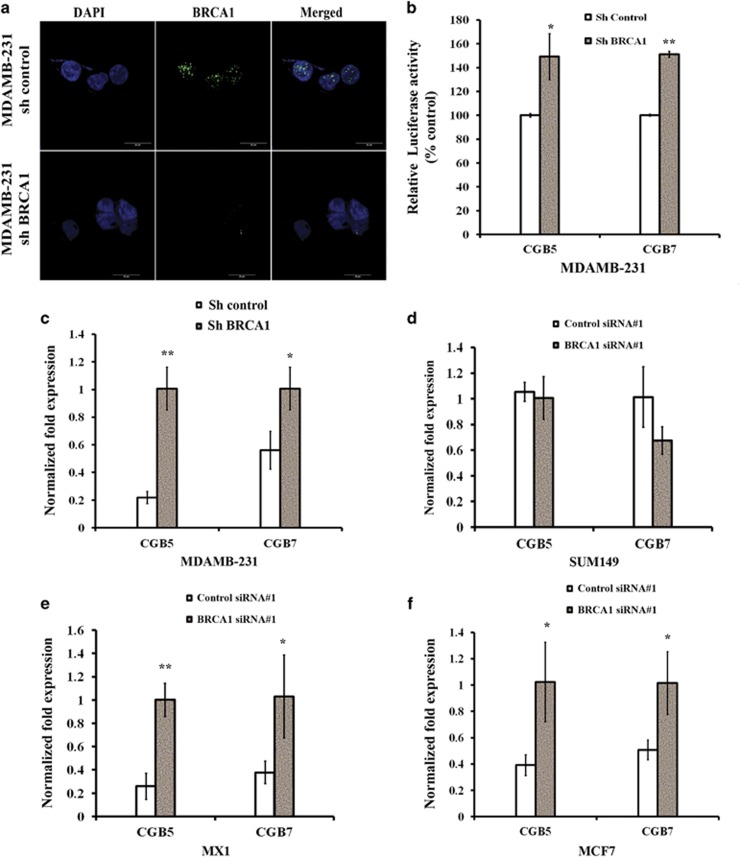
Loss of BRCA1 transcriptionally induces β-hCG expression in different breast cancer cells. (**a**) Immunoflourescence analysis of stable knockdown of BRCA1 in MDAMB-231 cells. (**b**) Relative Luciferase activity of CGB5 and CGB7 upon stable knockdown of BRCA1 in MDAMB-231. Stable clones of control shRNA transfected MDAMB-231 cells served as control. Relative luciferase units were expressed as percentage of control. (**c**) qRT-PCR analysis of CGB5 and CGB7 expression upon stable knockdown of BRCA1 in MDAMB-231. Expression was normalized to control shRNA transfected stable MDAMB-231. qRT-PCR analysis of CGB5 and CGB7 expression in (**d**) SUM149 (**e**) MX1 and (**f**) MCF7 upon silencing BRCA1. Expression was normalized to control siRNA transfected SUM149, MX1 and MCF7, respectively.

We analyzed the mRNA expression of the isoforms of β-hCG (CGB5 and CGB7) in a panel of breast, ovarian and prostate cancer cell lines and found that breast cancer cells harboring mutations in BRCA1 as shown in parenthesis, HCC1937 (5382insC), SUM149 (2288delT) and MX1 (33636delGAAA) express high levels of β-hCG, whereas β-hCG expression is barely detectable in cell lines possessing wild-type BRCA1 (MCF7, SKBR3, T47D, DU145, and OVCAR8) (Figure 1a). The levels of secreted β-hCG protein were also at least about fourfold higher in HCC1937 and SUM149 than MDAMB-231, MCF7 or OVCAR8 (Figure 1b). The above observations indicate that the expression of β-hCG might be limited to a few cancers which could be BRCA1 mutated. To check the influence of BRCA1 on β-hCG expression, we analyzed the expression of β-hCG in wild-type BRCA1 reconstituted HCC1937 (HCC1937/wt BRCA1). Stable reconstitution with wild-type BRCA1 in HCC1937 cells significantly reduced the expression of β-hCG (Figures 1c and d, and Supplementary Figure S1a). Further, we analyzed whether knockdown of BRCA1 in HCC1937/wt BRCA1 could rescue the expression of β-hCG. As expected, β-hCG expression was induced in HCC1937/wt BRCA1 upon BRCA1 knockdown using two different siRNA oligos (Figure 1e and Supplementary Figure S1c) whereas knockdown of mutated BRCA1 in HCC1937 has no effect on expression of β-hCG (Figure 1f and Supplementary Figure S1e). Knockdown of BRCA1 in HCC1937 and HCC1937/wt BRCA1 was confirmed using qRT-PCR, Immunoflourescence and Immunoblotting (Supplementary Figures S1b and d and S2a–c). Further, to check whether BRCA1 has any transcriptional regulation over β-hCG, we analyzed the presence of BRCA1 in the promoter of β-hCG by ChIP assay. Surprisingly, the wild-type, but not the mutant BRCA1 protein, is recruited to the promoter of β-hCG (Figures 1g and h). Stable knockdown of BRCA1 in MDAMB-231 has been confirmed using immunoflourescence (Figure 2a). Further, luciferase activity of both the isoforms of β-hCG was significantly induced upon stable knockdown of BRCA1 in MDAMB-231 (Figure 2b). It is evident that BRCA1 represses the expression of β-hCG and when BRCA1 is mutated/defective, the expression of β-hCG increases as the regulatory hold of BRCA1 on the β-hCG promoter is relieved.

### Knockdown of BRCA1 induces β-hCG in different breast cancer cell lines

Next, we analyzed whether knockdown of BRCA1 could induce β-hCG in BRCA1 wild-type breast cancer cells. BRCA1 knockdown has been confirmed by qRT-PCR and Immunoflourescence in different cell lines (Supplementary Figures S3a, c, e and S4a–c). Silencing of BRCA1 in MDAMB-231 induced the expression of β-hCG (Figure 2c). SUM149 has no effect on expression of β-hCG however, in MX1 cells, β-hCG is increased upon BRCA1 knockdown (Figures 2d and e and Supplementary Figures S3b and d). This could be attributed to the different functional isoforms of BRCA1 expressed in MX1. It is not limited to triple negative breast cancer cells, as β-hCG expression was induced in MCF7 upon BRCA1 knockdown (Figure 2f and Supplementary Figure S3f).

### β-hCG expression is induced in BRCA1 conditional knockout mice model

**Figure 3:**
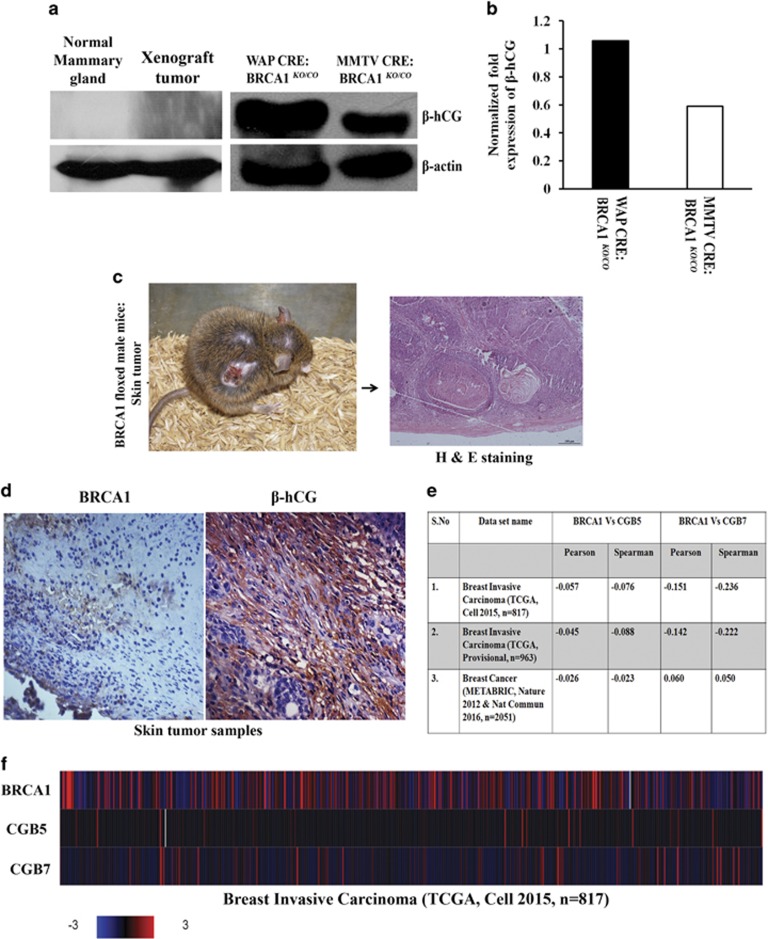
Loss of BRCA1 induces β-hCG expression *in vivo.* (**a**) Western blot analysis of β-hCG expression in normal mammary gland, xenograft tumor developed using MDAMB-231 cells and mouse mammary tumor of BRCA1 floxed conditional knockout mouse tissue generated from *WAP-Cre*; *BRCA1*^*KO/CO*^ and *MMTV-Cre*; *BRCA1*^*KO/CO*^ animals. (**b**) Adjacent panel represents the quantitation of western blot of *WAP-Cre*; *BRCA1*^*KO/CO*^ and *MMTV-Cre*; *BRCA1*^*KO/CO*^. (**c**) BRCA1 floxed male mouse showing the skin tumor. Right panel shows H & E staining of tumor region. (**d**) IHC analysis of β-hCG and BRCA1 in skin tumor of BRCA1 floxed mouse tumor tissues. (**e**) Table showing the pearson and spearman correlation values between BRCA1 vs CGB5 and BRCA1 vs CGB7 from three different human breast cancer dataset. (**f**) Heat map showing the expression levels of BRCA1, CGB5 and CGB7 from breast invasive carcinoma dataset (TCGA, cell 2015).

Having determined the role of BRCA1 in regulating β-hCG *in vitro*, we looked for similar conditions *in vivo*. To address this, two BRCA1 conditional knockout mice models (*WAP-Cre*; *BRCA1*^*KO/CO*^ and *MMTV-Cre*; *BRCA1*^*KO/CO*^) were developed as reported in our previous study.^18^ Mammary tumor formation in *WAP-Cre*; *BRCA1*^*KO/CO*^ was confirmed by NMR bio imager. As expected, a high expression of β-hCG was observed in breast tumor tissues of BRCA1 conditional knockout *WAP-Cre*; *BRCA1*^*KO/CO*^ mice. β-hCG was found to be overexpressed in *WAP-Cre*; *BRCA1*^*KO/CO*^ more than *MMTV-Cre*; *BRCA1*^*KO/CO*^ which indicates the tissue specific tumorigenicity of β-hCG (Figures 3a and b). Normal mammary gland and tumor samples from xenograft developed using human breast cancer cell line, MDAMB-231 did not express β-hCG (Figure 3a). In *WAP-Cre*; *BRCA1*^*KO/CO*^, the tumor was developed only after three pregnancies^17^ whereas MMTV developed tumors even without pregnancy. Probably, the exposure to pregnancy-induced hCG has a tumorigenic effect in *WAP-Cre*; *BRCA1*^*KO/CO*^ animals.

Interestingly, one of the BRCA1-floxed male mice developed spontaneous basal cell carcinoma (BCC) of the skin (Figure 3c). BRCA1 mutations in exon 11 were not detected in the tumor samples (Supplementary Figures S5a and b). However, reduction in the expression of BRCA1 was observed (Figure 3d). In addition, β-hCG expression is also induced in the BCC samples (Figure 3d). Since brca1 exon 11 is floxed by “loxP”, a truncation of the mRNA or generation of Brca1 11 splice variants might have occurred which resulted in loss of genomic stability and led to tumorigenesis. Genetically modified animals having floxed genes may develop spontaneous, non-targeted tumors due to reduction in the gene expression as a result of the presence of “loxP” sites in their genome. All the above observations clearly indicate that the loss of BRCA1 induces β-hCG in breast cancers. Further, human breast cancer tissue samples (*n*=3831) were analyzed using three cBioportal TCGA data sets, in which the expression levels of CGB5, CGB7 and BRCA1 were available. All the three data sets have shown significant inverse correlation between CGB5 and BRCA1 and two out of three data sets have shown significant inverse correlation between CGB7 and BRCA1 as analyzed by Pearson and Spearman correlation (Figure 3e). The expression levels of CGB5, CGB7 and BRCA1 from breast invasive carcinoma study (TCGA, Cell 2015) is represented as heat map (Figure 3f).

### β-hCG induces metastatic ability of cancer cells through EMT in BRCA1 defective cells

**Figure 4:**
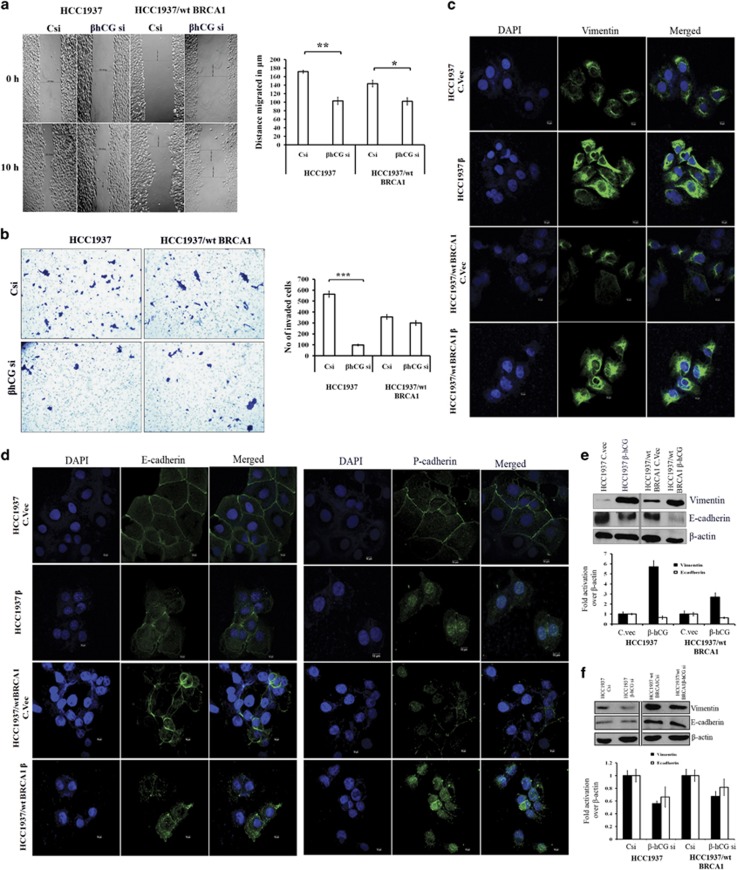
β-hCG induces migration, invasion and EMT. (**a**) Wound healing assay in HCC1937 and HCC1937/wt BRCA1 upon silencing the endogenous β-hCG expression by transfecting with β-hCG siRNA. “Csi” represents scrambled siRNA and “β-hCG si” represents β-hCG siRNA. Graph represents the distance migrated by cells in μm. (**b**) Invasion assay in HCC1937 and HCC1937/wt BRCA1 upon silencing the endogenous β-hCG expression. Graph represents the total number of invaded cells. (**c**) Immunoflourescence of Vimentin in HCC1937 control vector transfected (HCC1937 C.vec), HCC1937 β-hCG transfected (HCC1937 β), HCC1937/wt BRCA1 control vector transfected (HCC1937/wt BRCA1 C.vec) and HCC1937/wt BRCA1 β-hCG transfected (HCC1937/wt BRCA1 β) cells. DAPI was used as nuclear stain. (**d**) Immunoflourescence of E-cadherin and P-cadherin in HCC1937 C.vec, HCC1937 β, HCC1937/wt BRCA1 C.vec and HCC1937/wt BRCA1 β. (**e**) Western blot analysis of the Vimentin and E-cadherin upon overexpression of β-hCG in HCC1937 and HCC1937/wt BRCA1 and (**f**) upon silencing the endogenous β-hCG expression in HCC1937 and HCC1937/wt BRCA1.

**Figure 5:**
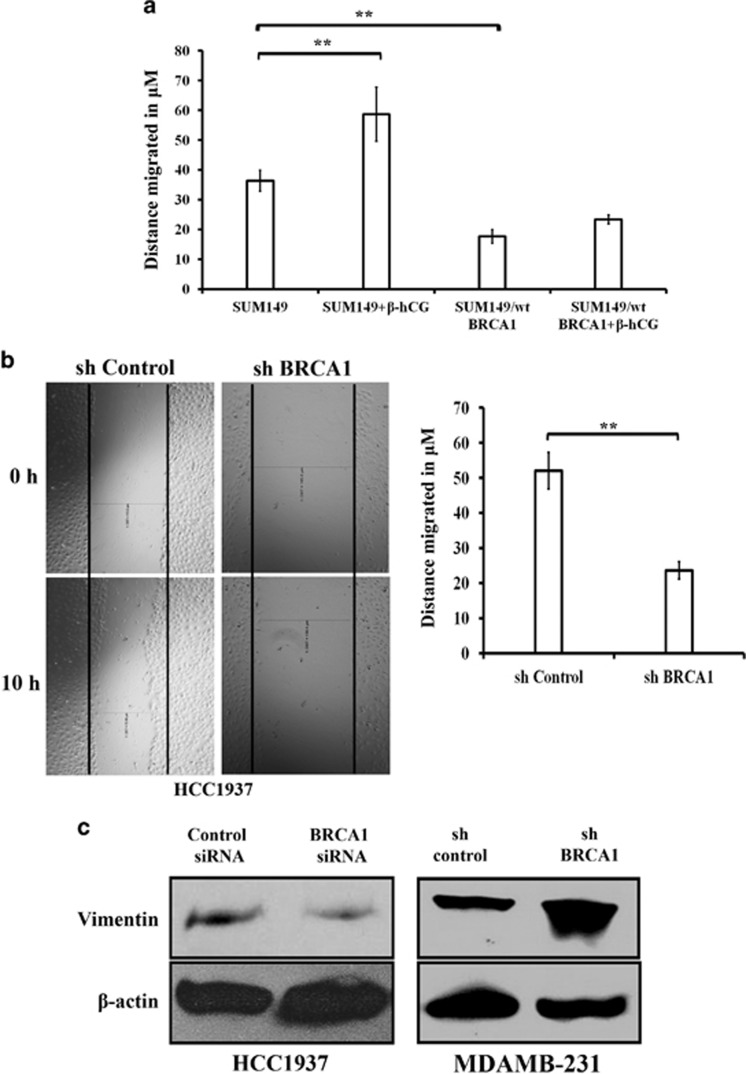
Mutant BRCA1 augments β-hCG induced migration in BRCA1 defective cells. (**a**) Wound healing assay of SUM149 and SUM149/wt BRCA1 upon supplementing exogenous β-hCG. Graph represents the distance migrated by cells in μm. (**b**) Wound healing assay of sh control and sh BRCA1 silenced HCC1937. Graph on the right panel represents the distance migrated by cells in μm. (**c**) Western blot analysis of the vimentin upon silencing BRCA1 in HCC1937 and MDAMB-231 by siRNA and shRNA respectively. Control siRNA and sh control served as control for HCC1937 and MDAMB-231, respectively.

Due to the high expression of β-hCG, we hypothesized that β-hCG might induce tumorigenicity in BRCA1 mutated breast cancer. To test this, we assessed the functional significance of β-hCG in both the cell lines (HCC1937 and HCC1937/wt BRCA1) by analyzing the migratory and invasive capacity. Upon knockdown of endogenous β-hCG by siRNA, the cells showed a significant reduction in migration and invasion which was more pronounced in HCC1937 (Figures 4a and b and Supplementary Figure S6a). Further, SUM149 cells were stably reconstituted with wild-type BRCA1 and the migratory ability of these cells were analyzed in the presence or in the absence of β-hCG. Reconstitution of wild-type BRCA1 in SUM149 (SUM149/wt BRCA1) reduced the migratory ability of the cells. Exogenous supplementation of β-hCG induced migration of SUM149 cells, however exogenous supplementation of β-hCG in SUM149/wt BRCA1 has no significant effect on the migratory ability of the cells (Figure 5a), clearly confirming the role of β-hCG in inducing metastasis specifically in BRCA1 defective cells. Since migration and invasion has been induced more specifically in BRCA1 defective cells by β-hCG, we checked whether EMT is also promoted in these cells. In HCC1937, the expression of mesenchymal marker, vimentin is significantly increased at mRNA and protein level both by overexpression and by exogenous supplementation of β-hCG (Figure 4c and Supplementary Figures S6b and c). In HCC1937/wt BRCA1, though the levels of vimentin at the mRNA level were decreased, vimentin remained overexpressed at the protein level both by exogenous supplementation and by overexpression of β-hCG, though the increase was lower than HCC1937. In the presence of β-hCG, the epithelial marker E-cadherin is decreased in both the cell lines (Figures 4d and e). A shift in the localization of P-cadherin from cytoplasmic membrane to nucleus was observed in the presence of β-hCG (Figure 4d). These changes on vimentin and E-cadherin expression were reversed upon inhibition of β-hCG by siRNA and were more pronounced in HCC1937 (Figure 4f and Supplementary Figure S6d). All these observations clearly indicate that β-hCG induces the migratory and invasive potential by promoting the process of EMT more specifically in BRCA1 defective than BRCA1 wild-type breast cancer cells.

**Figure 6:**
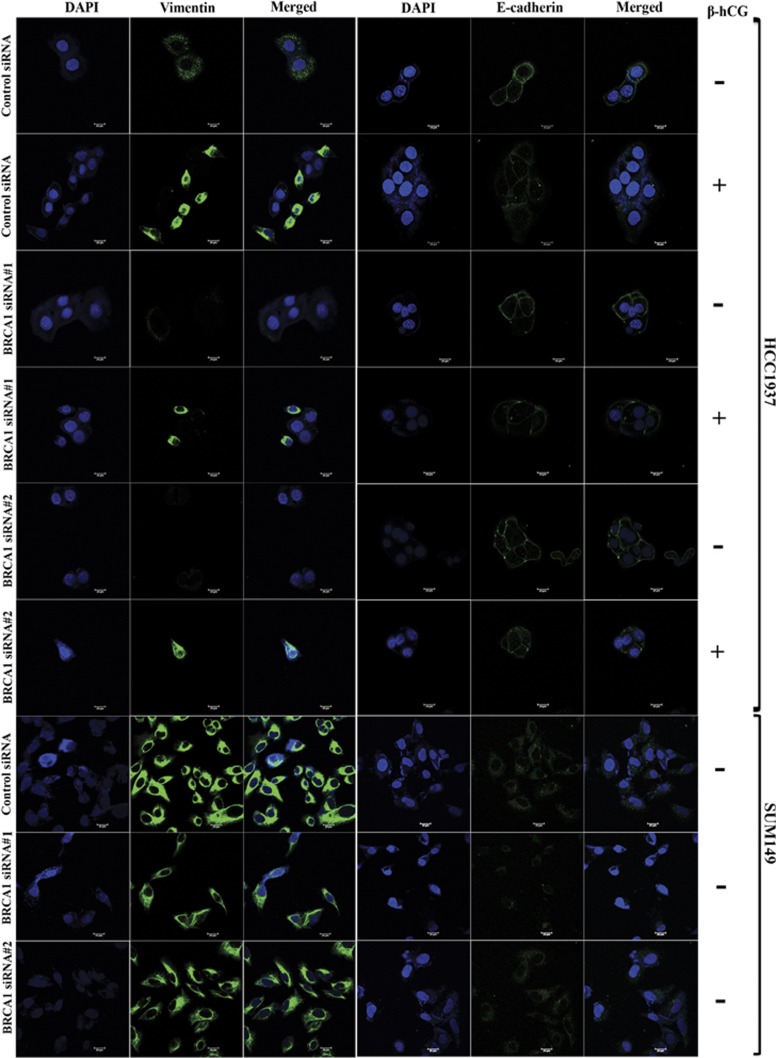
Mutant BRCA1 augments β-hCG induced EMT in BRCA1 defective cells. Immunoflourescence analysis of Vimentin and E-cadherin in control silenced (control siRNA) and BRCA1 silenced (BRCA1 siRNA#1 and BRCA1 siRNA#2) HCC1937 and SUM149 cells. “−” represents β-hCG unsupplemented cells and “+” represents exogenous β-hCG supplementated cells.

Further to understand the role of mutant BRCA1 in inducing migration, stable cells upon knocking down mutant BRCA1 has been developed in HCC1937 and the migratory ability of these cells has been analyzed. Inhibition of mutant BRCA1 severely hampers the migratory potential of these cells in HCC1937 (Figure 5b). Further to understand the involvement of mutant BRCA1 in EMT, expression of vimentin and E-cadherin was analyzed in mutant BRCA1 silenced HCC1937 and SUM149. Vimentin is reduced upon silencing of mutant BRCA1 by siRNA in HCC1937 and SUM149, whereas silencing of wild-type BRCA1 in MDAMB-231 by shRNA induced the expression of vimentin (Figures 5c and 6). As expected, exogenous supplementation of β-hCG in mutant BRCA1 silenced HCC1937 induced the expression of vimentin; however the induction was lower than β-hCG supplemented control silenced HCC1937 cells (Figure 6). Though no drastic changes were observed in E-cadherin upon silencing mutant BRCA1 in HCC1937 and SUM149, exogenous supplementation of β-hCG reduced the expression of E-cadherin in mutant BRCA1 silenced HCC1937, however the reduction was lower than β-hCG supplemented control silenced HCC1937 cells (Figure 6). All the above observations clearly demonstrate the significance of mutant BRCA1 in β-hCG induced metastatic potential of these cells.

### β-hCG overexpression increases stemness in BRCA1 mutant but not in wild-type cells

**Figure 7:**
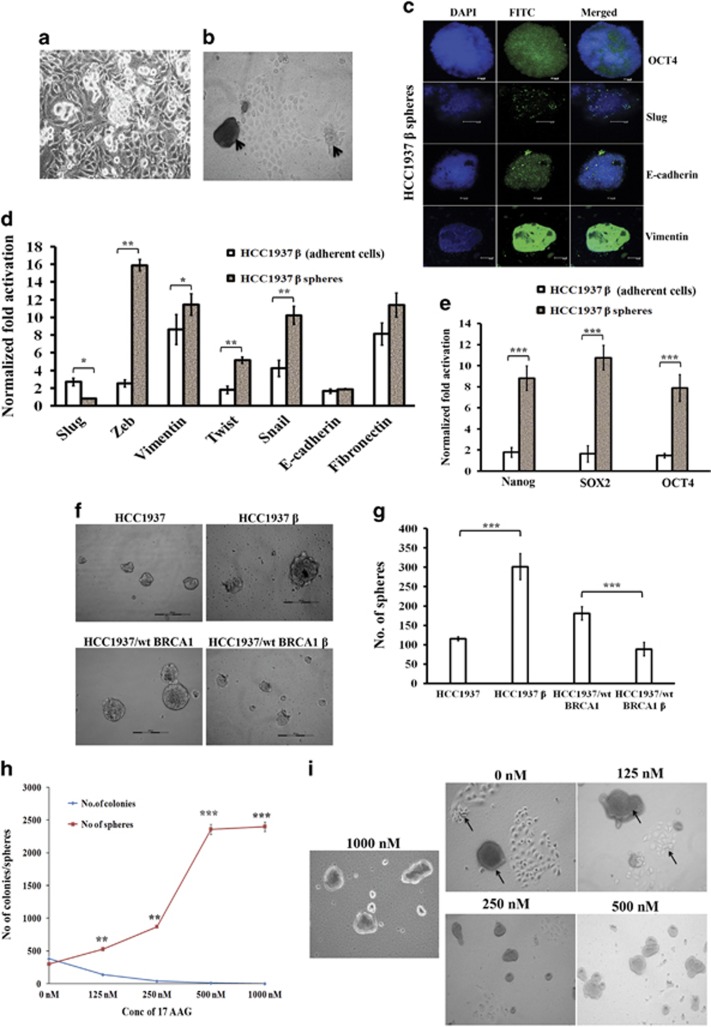
β-hCG induces stemness in HCC1937. (**a**) Sphere formation upon β-hCG overexpression in HCC1937 cells. (**b**) Arrows indicate the formation of spheres from adherent cells. (**c**) Immunoflourescence analysis of OCT4, Slug, E-cadherin and Vimentin in spheres derived from HCC1937 β. (**d**) qRT-PCR analysis of EMT markers (Slug, Zeb, Vimentin, Twist, Snail, E-cadherin and fibronectin) in the spheres and adherent cells of β-hCG overexpressed HCC1937. (**e**) qRT-PCR analysis of stem cell markers (Nanog, SOX2 and OCT4) in the spheres and adherent cells of β-hCG overexpressed HCC1937. (**f**) Mammosphere forming ability in non-adherent plates on day 4 in HCC1937, HCC1937/wt BRCA1, HCC1937 β and HCC1937/wt BRCA1 β. (**g**) Mammosphere with a minimum size of 50 μm from HCC1937, HCC1937/wt BRCA1, HCC1937 β and HCC1937/wt BRCA1 β was counted manually. All the experiments were done in triplicates. (**h**) Number of spheres (>50 μm in size) and number of adherent colonies (>around 50 cells/colonies) were counted manually in β-hCG overexpressing HCC1937 after treating with indicated doses of drug, 17AAG for 72 h. (**i**) Representative images of colony forming and sphere forming ability of β-hCG overexpressing HCC1937 (HCC1937 β) after treating with indicated doses of 17AAG for 9 days. All error bars in the graphs represent s.d.

*In vitro* sphere formation analysis is the key indicator representing the stem cell nature of cancer cells. We have observed a novel event in the β-hCG overexpressing HCC1937 (HCC1937 β) and SUM149 (SUM149 β) but not in β-hCG overexpressing HCC1937/wt BRCA1 (HCC1937/wt BRCA1 β) cells, where HCC1937 β and SUM149 β cells were able to form spheres from the monolayer culture when grown for 4–5 days in an adherent cell culture plate in medium supplemented with 10% FBS even in the absence of any growth factor supplements. When cells were seeded in limiting dilution, the individual cells formed colonies, along with that it also formed spheres with a size upto about 250 μm in diameter (Figures 7a and b and Supplementary Figure S7a). We could subculture the spheres to generate both adherent cells and spheres again. We observed that the spheres have stemness, expressing high levels of EMT and stem cell markers (Figures 7c–e and Supplementary Figures S7b–d). Three-day culture in non-adherent plates resulted in the formation of mammospheres, the number and size of the spheres were lower in HCC1937/wt BRCA1 β than in HCC1937 β (Figures 7f and g). Since 17 AAG, an HSP90 inhibitor is being evaluated in clinical trials for targeting BRCA1 defective tumors, we analyzed whether these spheres could be targeted by 17 AAG. As the concentration of 17AAG was increased, the number of adherent colonies decreased as expected, but interestingly, the number of spheres were increased (Figures 7h and i). This might be attributed to the increase in the resistance of the cells to the chemotherapeutic drugs. All these data clearly indicate that the process of β-hCG mediated stemness and EMT has been induced in HCC1937 and SUM149 β cells which harbors mutant BRCA1 but not in HCC1937/wt BRCA1 β which has wild-type BRCA1.

### β-hCG induces tumorigenesis in BRCA1 defective cancer cells via TGFβRII signaling

**Figure 8:**
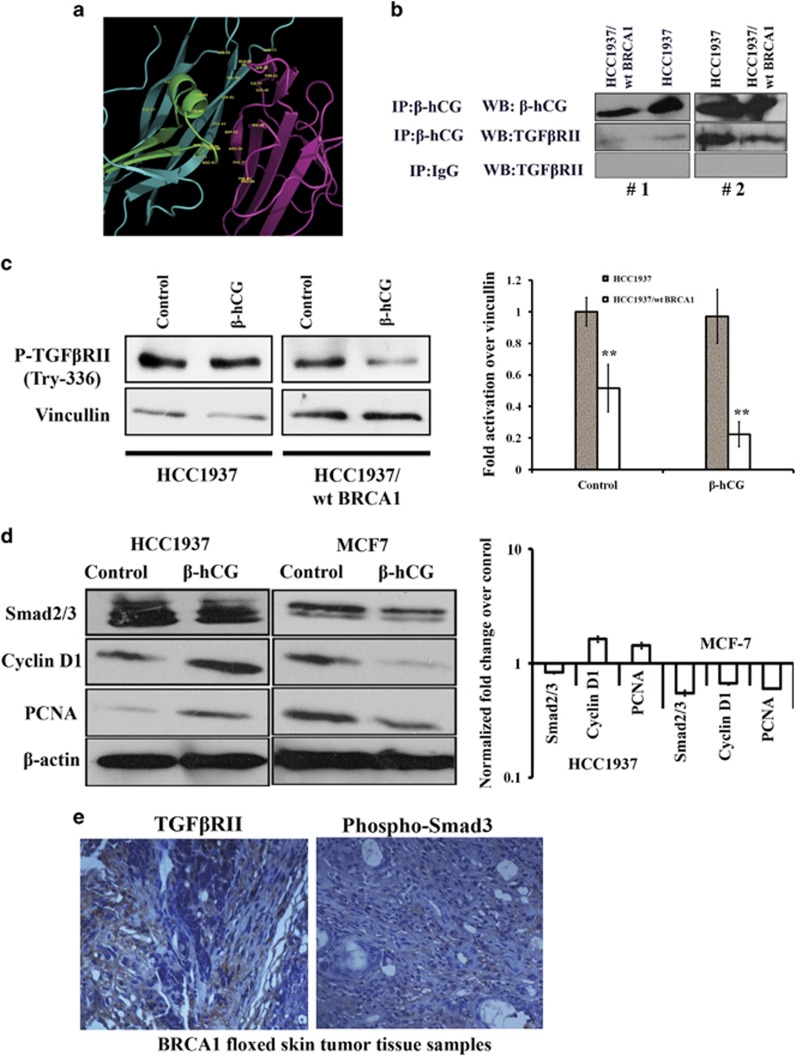
β-hCG acts via TGFβRII in BRCA1 mutated breast cancer cells. (**a**) *In silico* analysis of interaction between β-hCG and TGFβRII. (**b**) Western blot of β-hCG and TGFβRII from samples immunoprecipitated with anti-β-hCG or anti-IgG antibody in HCC1937 and HCC1937/wt BRCA1. IgG was used as negative control. #1 and #2 represents the repetition of the experiment. (**c**) Western blot analysis of P-TGFβRII (Try 336) upon treating HCC1937 and HCC1937/wt BRCA1 cells with β-hCG. (**d**) Western blot analysis of Smad2/3, Cyclin D1 and PCNA upon supplementation of β-hCG in HCC1937 and MCF7. Right panel shows the quantification of the blot. (**e**) IHC analysis of TGFβRII and Phospho-Smad3 in the mouse skin tumor of BRCA1 floxed tumor tissue samples.

**Figure 9:**
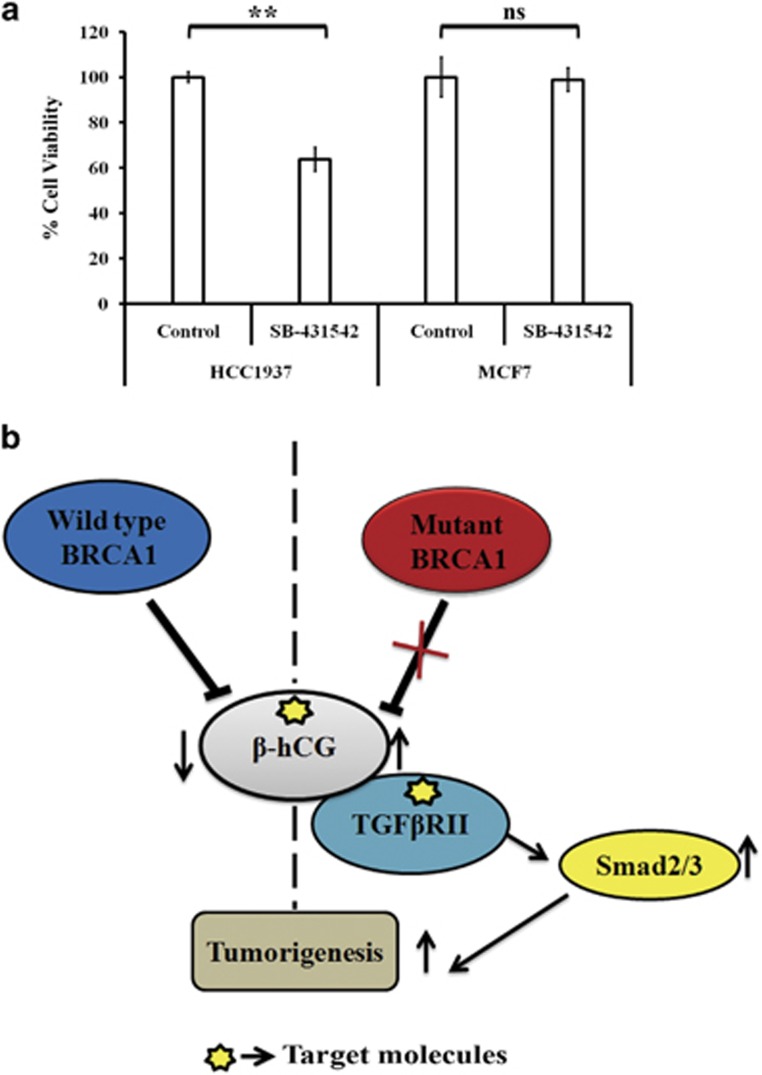
Regulation between β-hCG, BRCA1 and TGFβRII in breast cancer. (**a**) Cell viability assay (MTS) of HCC1937 and MCF7 cells upon treating with TGFβRII inhibitor, SB-431542 for 48 h. (**b**) Summary of regulation between β-hCG and BRCA1 and possible tumorigenesis in BRCA1 defective breast tumors. *indicates the possible targets for effective inhibition of tumorigenesis in BRCA1 mutated cancer cells.

Since β-hCG is secreted by the BRCA1 defective cells, we looked for the possible receptor through which it exerts its tumorigenic effect. We confirmed a high expression of Luteinizing hormone/choriogonadotropin receptor (LHCGR) in TGCA data set for breast cancer and in a panel of breast cancer cell lines, however LHCGR was found to be expressed irrespective of β-hCG expression status (Supplementary Figures S8a–c). Also, full length hCG and β-hCG are functionally different in these cell lines as supplementation of full length hCG induced the cells to adhere even in the absence of serum probably by acting through LHCGR but a similar effect was not observed in the case of β-hCG (Supplementary Figure S8d). Also, LHCGR is expressed by the BRCA1 conditional knockout mouse skin tumor tissue (Supplementary Figure S8e). All these observations indicate that the expression of LHCGR could be independent of β-hCG at least in these tissues. Since β-hCG belongs to a group of cysteine knot family of proteins (which also includes TGFβ, VEGF, PDGF and so on), in which the members of the family can share their receptors,^19^ we checked whether β-hCG could mediate signaling through TGFβRII. Here, we identified that β-hCG binds to TGFβRII in breast cancer cells (Figures 8a and b). In breast cancer cell lines, Phospho-TGFβRII (Try 336) was analyzed upon supplementation of β-hCG. P-TGFβRII was maintained in HCC1937 supplemented with β-hCG whereas reduction was observed in the HCC1937/wt BRCA1 cell line (Figure 8c). Concurrently, expression of Smad2/3 was observed upon supplementation of β-hCG in HCC1937. This indicates that TGFβRII mediated Smad signaling could be induced by β-hCG in BRCA1 mutated cells. Further, the proliferation marker, PCNA was induced in HCC1937 but reduced in BRCA1 wild-type condition upon supplementation of β-hCG. Smad3 was known to induce Cyclin D1 and was found to be induced in the presence of β-hCG in HCC1937 (Figure 8d). TGFβRII receptor and Phospho-Smad3 expression was correspondingly observed in *WAP-Cre*; *BRCA1*^*KO/CO*^ skin cancer tissues (Figure 8e). Further to confirm, chemical inhibitor for TGFβRII (SB-431542) was used to downregulate TGFβRII expression and the effect on cell proliferation has been analyzed in BRCA1 mutant HCC1937 and BRCA1 wild-type MCF7. We show that inhibition of TGFβRII has severe effect on cell proliferation of BRCA1 mutant β-hCG expressing HCC1937 whereas such effect was not observed in BRCA1 wild-type β-hCG non-expressing MCF7 (Figure 9a). All these observations clearly show the tumorigenic role of β-hCG in BRCA1 defective condition could be mediated via TGFβRII. Analysis of TCGA data sets by Oncomine and cBioPortal, showed that pancreas/breast/bladder cancers present significant amplification of CGB5 (Supplementary Figures S9a and b). BRCA1 mutation is also reported in pancreatic/bladder cancers which could be the reason why some of these tumors show β-hCG expression in TCGA data set.^20, 21, 22, 23^ All together our results clearly show the tumorigenic role of β-hCG in BRCA1 defective cells which could be mediated through TGFβRII.

## Discussion

We report a high expression of β-hCG in BRCA1 mutated breast cancer cell line which was demonstrated *in vitro* and *in vivo*. When wild-type BRCA1 is reconstituted in BRCA1 mutant cells, the expression of β-hCG reduces. Also, wild-type but not mutant BRCA1 transcriptionally regulates the expression of β-hCG and knockdown of wild-type BRCA1 induces β-hCG in different breast cancer cell lines. Further β-hCG is expressed in BRCA1 deficient cancer cells irrespective of triple negative status. TCGA analysis using cBioportal data sets confirms the inverse correlation between the levels of CGB5, CGB7 and BRCA1 in human breast cancer tissue samples. Probably this is the reason why only some of the breast cancer tissues express β-hCG^24, 25^ in a BRCA1 defective or BRCA1 deficient such as BRCA1 hyper-methylated condition. Though a regulation between BRCA1 and ER-α has been reported earlier and has been hypothesized as one of the causes for predisposition of BRCA1 mutated cancers to breast tissue, the tumorigenic effect could not be attributed to ER-α as most of the BRCA1 defective cancers are also triple negative. Since β-hCG is upregulated in BRCA1 defective but not in wild-type condition, β-hCG would have a major role in tumorigenesis in BRCA1 defective cancers.

BRCA1 mutation has been recently reported to promote migration and invasion by interacting with Ezrin-Radixin-Moesin proteins in the plasma membrane.^26^ β-hCG has also been demonstrated to promote metastasis in prostate cancer cells. We report a high metastatic potential of β-hCG in BRCA1 defective cancers as inhibition of β-hCG can result in 15% and 55% more reduction in migration and invasion respectively in BRCA1 mutant than in wild-type cancer cells. In accordance with this, EMT markers were overexpressed in the presence of β-hCG in BRCA1 mutant than in wild-type cells. Further, mutant BRCA1 is critical for the induction of β-hCG induced metastasis in BRCA1 defective cells. Probably, it could be attributed to the gain of function of mutant BRCA1. An interesting phenomenon was also observed in β-hCG overexpressing BRCA1 mutant but not in wild-type cell line where the cells could form spheres even in adherent plates and expresses the markers of stemness and EMT. Downregulation of IL3, IL13R, TNF12 and TNF10 was observed in BRCA1 mutated compared to BRCA1 wild-type cells as assessed by microarray analysis (data not shown). This could be probably due to the immune suppressive activity of β-hCG.^27, 28^ This clearly shows that β-hCG could be one of the key players in inducing aggressiveness particularly in BRCA1 mutated triple negative breast cancers.

Since β-hCG is itself secreted by BRCA1 mutated cancers, it could have an autocrine effect in inducing tumorigenesis of such cancers. It has been noticed that β-hCG expressed by these cancer cells may not induce growth signal through LHCGR as there was no correlation observed between LHCGR and β-hCG in breast cancer cells. However, it was observed that β-hCG could directly interact with TGFβRII and induces signaling by activating Smad3 in BRCA1 defective β-hCG overexpressing cells and inhibition of TGFβRII hampers the cell proliferation in BRCA1 defective cells. Mechanistically, loss of transcriptional inhibition by BRCA1 on β-hCG could be an important event in the tumorigenesis of BRCA1 mutated breast tumors (Figure 9b). Considering the fact that β-hCG is an immune suppressor and is overexpressed in BRCA1 defective cancer cells, inhibiting β-hCG could be a better treatment strategy with immense clinical application than using immune modulators for reducing the growth of such cancers. Passive immunization with β-hCG antibodies and/or β-hCG antibodies tagged with anticancer agents may therefore have exciting clinical potential and may give excellent lead for clinicians designing future trials for treating BRCA1 defective cancers.

## Materials and methods

### Cell lines

Breast (MCF7, MDAMB-231, SKBR3 and T47D), prostate (DU145) and ovarian (OVCAR8) cancer cell lines were obtained from ATCC. SUM149 was a kind gift from Dr. Mieke Schutte, Erasmus MC, University Medical Centre, Rotterdam, The Netherlands. MX1 was obtained as a kind gift from Dr. Cathrin Dressler, Laser- und Medizin-Technologie GmbH, Berlin, Germany. HCC1937 and HCC1937/wt BRCA1 were obtained as gift from Dr. Grant McArthur, Peter MacCallum Cancer Centre, VIC, Australia. All the cell lines were grown in DMEM except SUM149 which was grown in Ham’s F12 and HCC1937, HCC1937/wt BRCA1 was grown in RPMI. All cell lines were maintained in media supplemented with 10% FBS at 37 °C in a CO_2_ incubator.

### TCGA data analysis

cBioportal for cancer genomics was utilized to analyze the expression of CGB5, CGB7 and BRCA1 in breast cancer tissues samples (http://www.cbioportal.org/). The Cancer Genome Atlas research network (TCGA) data set (*n*=27) (http://cancergenome.nih.gov/) for different types of cancer was analyzed with respect to normal tissue samples for the CGB5 expression with the help of Oncomine.

### Real time PCR, Semi-quantitative PCR and ChIP

Total RNA was isolated using High Pure RNA isolation kit (Roche, 11828665001), and quantified using Nanodrop (Nanodrop, Thermo fisher Scientific). A total RNA of 1000 ng was used for cDNA synthesis (4368813, Life technologies) as per the manufacturer’s protocol. Real time PCR was done in a Biorad CFX96 using syber green (KM4101, Kappa biosciences) as per the manufacturer’s protocol. For semi-quantitative PCR, Green GoTaq polymerase system (M791B, Promega) was used as per the manufacturer’s protocol and the PCR amplified product was resolved on 1% agarose, viewed and imaged using gel doc system (Biorad, Model: Universal Hood II).

For ChIP experiments, 90% confluent cells in 100 mm dishes were cross linked with 37% formaldehyde. The cells were lysed and immunoprecipitated as per manufacturer’s protocol (1710085, Millipore) using anti-BRCA1 or IgG antibody (sc-2025, Santa Cruz Biotechnology). The purified DNA fragments were amplified by real time PCR or semi-quantitative PCR as mentioned before using specific primers.

### Immunoflourescence, immunoblotting and immunoprecipitation

Immunoflourescence and immunoblotting were carried out as described earlier.^29^ For immunoprecipitation, cells were lysed with NP40 lysis buffer and 1 mg of total protein was immunoprecipitated with IP specific antibodies for 1 h at 4 °C. Protein lysate and protein A/G beads (17-5280-01, GE Healthcare) were mixed and kept at shaking overnight at 4 °C. The samples were boiled with lammeli sample buffer (161-0737, Biorad) containing β-mercaptoethanol for 5 min and the supernatant was resolved on 10% SDS-polyacrylamide gel and immunoblotting was done with help of immunoprecipitating antibody (input) or non-immunoprecipitating antibody.

### siRNAs, expression constructs

For siRNA transfection, 80 pmoles of β-hCG siRNA (sc-39540, Santa Cruz Biotechnology) and 4 nmoles of BRCA1 siRNA (4507241, Eurogentec), BRCA1 siRNA (sc-29219, Santa Cruz Biotechnology) and the transfection reagent, IcaFectin 442 (Eurogentec, 442-500) were used as per manufacturer’s instructions. From 80 pmoles to 4 nmoles of scrambled siRNA (1027280, Qiagen) was used as control. β-hCG plasmid (16574, Addgene) and BRCA1 plasmid (14999, Addgene) was obtained as per the guidelines of Addgene. For stable transfection, the cells were selected using 200 μg/ml of G418 (10131-035, Gibco) or Puromycin (P8833, Sigma-Aldrich) for 45 days after transfecting β-hCG or BRCA1 or sh control or sh BRCA1 plasmids. Fresh medium with G418 and/or puromycin was replaced every third day.

### Sphere formation

After HCC1937 and SUM149 β cells were allowed to grow for 4–5 days in an adherent cell culture plate, the spheres were then collected by centrifuging the supernatant at 1000 r.p.m. at RT. The spheres were washed twice with PBS and seeded in a serum free condition for 5–8 days. For mammosphere generation, cells were seeded in low attachment 24-well multidish (Corning, 3474) at a density of 3000 cells/well and grown in MEBM medium supplemented with hydrocortisone (Sigma-Aldrich, H6909), Insulin (Sigma-Aldrich, 19278), bFGF (BD Biosciences, 354060), EGF (BD Biosciences, 354052), B27 (Gibco, 17504-044) and Heparin (Sigma-Aldrich, H3149) for 4–7 days. The number of mammospheres was counted manually in the entire field and the size of the mammospheres was measured using vernier scale and photographed using phase contrast microscope.

### Transgenic mouse model

WAP-Cre mice (STOCK 01XA8, B6.Cg-Tg (Wap-Cre) 11738Mam), MMTV-Cre mice (STOCK 01XA9, B6.Cg-Tg (MMTV-Cre) FMam) and BRCA1 floxed mice (STOCK 01XC8 Brca1tm1Cxd), obtained from the NCI Mouse Repository at the National Cancer Institute (NCI), USA were used for the generation of *WAP-Cre*; *BRCA1*^*KO/CO*^ and *MMTV-Cre*; *BRCA1*^*KO/CO*^ conditional knockout mouse models. The experiments were performed as per the protocols mentioned in Institutional Animal Ethical Committee, Centre for Cellular and Molecular Biology (CCMB), Hyderabad, India. Detailed protocol is mentioned in our earlier report.^18^ A portion of tissue was used for protein isolation and the remaining tissue was fixed in 10% formalin and Paraffin Embedded Tissue (PET) sections were made using the protocol mentioned earlier.^18^

### Sequencing the exon 11 of Brca1 gene

Primer set B004/B005 is the main genotyping set and detects the 38 bp loxP insert upstream of Exon 11. Primer set B004/B006 is an extra control to ensure that spontaneous recombination has not occurred, deleting Exon 11. Details of primer pairs are provided in Table 1. BRCA1 exon 11 PCR products was eluted out from the gel using Gel extraction kit (Qiagen, Hilden, Germany) by following the manufacturer's instructions. Sequencing of all the samples were performed using an ABI 3730 Genetic Analyser automated DNA analyzer (PE Applied Biosystems, Foster City, CA) using sequence specific primers.

### Immunohistochemical analysis

Immunohistochemical analysis (IHC) was carried out as described previously.^30^ The slides were viewed and photographed under light microscope.

### Antibodies

Antibodies used for all the experiments were as follows: for immunoflourescence, β-hCG (ab9582, Abcam), Vimentin (5144 S, Cell signaling technology), E-cadherin (3195 S, Cell signaling technology), P-cadherin (sc-7893, Santa Cruz Biotechnology), BRCA1 (9010 S, Cell signaling technology) primary antibodies were followed by secondary antibodies conjugated with FITC (35552, Cell Signaling Technology). For immunoblotting, Vimentin (5144 S, Cell signaling technology), E-cadherin (3195 S, Cell signaling technology), β-actin (sc-47778, Santa Cruz Biotechnology). For immunoprecipitation: β-hCG (sc-271062, Santa Cruz Biotechnology), TGF beta Receptor II (ab78419, Abcam), normal IgG (sc-2025, Santa Cruz Biotechnology). ChIP: BRCA1 (A301-377 A, Bethyl laboratories). Immunohistochemical analysis: β-hCG (AM305-5M, Biogenex), BRCA1 (AR345-5 R, Biogenex).

### ELISA

Cells were seeded at a density of 1 × 10^5^ in 24-well plates and allowed to grow in the 10% media. After 24 h, the media was replaced with serum free media and at different time points 100 μl of conditioned media was collected and replaced with 100 μl of SFM. The presence of β-hCG in SFM was detected using β-hCG ELISA kit (ab100533, Abcam) as per the manufacturer’s protocol.

### Colony formation assay and MTS assay

For colony formation assay, the cells supplemented with or without β-hCG for 24 h or HCC1937 β cells were treated with 17 AAG for 72 h. Cells seeded at a density of 1000 cells/well in 6-well plate and the colonies were assessed after 14 days. Colonies were fixed with methanol: acetic acid (3:1) and stained with crystal violet. Colonies with a minimum of 50 cells were counted manually. For MTS assay, HCC1937 and MCF7 cells seeded at a density of 5000 cells in 96 well were treated with TGFβRII inhibitor, SB-431542 (S4317, Sigma-Aldrich) for 48 h and the cell viability was assessed as per manufacturer’s protocol (G5421, Promega).

### Statistical analysis

Statistical analysis was performed by one way ANOVA (Student's *t*-test). Experiments were performed in technical and biological replicates and the error bars are shown in s.d. *P*-value <0.05 was considered statistically significant. Statistical analysis for animal experiment was reported in our earlier report.^18^

## Publisher’s note

Springer Nature remains neutral with regard to jurisdictional claims in published maps and institutional affiliations.

## Figures and Tables

**Table 1:** Primers for sequencing the exon 11 of Brca1 gene

Primer name	Sequence (5′–3′)
BRCA1-B004	CTGGGTAGTTTGTAAGCATGC
BRCA1-B005	CAATAAACTGCTGGTCTCAGG
BRCA1-B006	CTGCGAGCAGTCTTCAGAAAG
