# Supplementary material for: BRCA1 regulation on β-hCG: a mechanism for tumorigenicity in BRCA1 defective breast cancer
Source: Oncogenesis. 2017 Sep 4;6(9):e376–. doi: 10.1038/oncsis.2017.75 (PMC5623901; doi:10.1038/oncsis.2017.75)
Supplement: Supplementary Information [file oncsis201775x1.doc]

**Supplementary Materials and Methods**

The primers used for all the experiments were listed as follows.

CGB7 forward 5'-CCTCCCTGGCCTTGTCTACTTCTC-3'

CGB7 reverse 5'-GCACCGTGGCCGAAGCAT-3'

CGB5 forward 5'-TGAGCCACTCCTGCGCCC-3'

CGB5 reverse 5'-CAGCCCCTGGAACATCTCCA-3'

GAPDH forward 5′-CAACTACATGGTTTACATGTTC-3′

GAPDH reverse 5′-GCCAGTGGACTCCACGAC-3′

BRCA1 forward 5'-AAGGTTGTTGATGTGGAGGAG-3'

BRCA1 reverse 5'-CAGAGGTTGAAGATGGTATGTTG-3'

Sox2 forward 5′-GGGAAATGGGAGGGGTGCAAAAGAGG-3′

Sox2 reverse 5′-TTGCGTGAGTGTGGATGGGGATTGGTG-3′

slug forward 5'-AGA TGC ATA TTC GGA CCCAC-3′

slug reverse 5'-CCT CAT GTT TGT GCA GGA GA-3′

snail forward 5'-AAT CGG AAG CCT AAC TAC AGC GAG-3′

snail reverse 5'-CCT TGG CCT CAG AGA GCT GG-3′

oct 4 forward 5'-CGCAAGCCCTCATTTCAC-3'

oct4 reverse 5'-CATCACCTCCACCACCTG-3'

nanog forward 5'-TGCCTCACACGGAGACTG-3'

nanog reverse 5'-GCTATTCTTCGGCCAGTT-3'

nestin forward 5'-CAGCTGGCGCACCTCAAGATG-3'

nestin reverse 5'-AGGGAAGTTGGGCTCAGGACTGG-3'

Twist forward 5'-GGACAAGCTGAGCAAGATTCAGA-3′

Twist reverse 5′-TCTGGAGGACCTGGTAGAGGAA-3′

zeb1 forward 5'-GGCAGAGAATGAGGGAGAAG-3'

zeb1 reverse 5'-CTTCAGACACTTGCTCACTACTC-3'

Vimentin Forward 5'GACAATGCGTCTCTGGCACGTCTT3'

Vimentin Reverse 5'TCCTCCGCCTCCTGCAGGTTCTT3'

Ecadherin Forward 5'GAAGGTGACAGAGCCTCTGGAT3'

Ecadherin Reverse 5'GATCGGTTACCGTGATCAAAATC3'

Fibronectin Forward 5′GAAGCTCTCTCTCAGACAACCA3′

Fibronectin Reverse 5′GCCCACGGTAACAACCTCTT3'

**Supplementary Figure Legends**

**Supplementary Figure S1: Expression of β-hCG upon BRCA1 silencing.** a) Immunoflourescence of β-hCG in HCC1937 and HCC1937/wt BRCA1. BRCAl silencing was confirmed by RT-qPCR in b) HCC1937 and d) HCC1937/wt BRCA1. Expression of CGB5 and CGB7 in BRCA1 silenced c) HCC1937 and e) HCC1937/wt BRCA1 by RT-qPCR. Expression was normalized to control scrambled siRNA transfected HCC1937 and HCC1937/wt BRCA1. Two different siRNAs, BRCA1 siRNA #1 and BRCA1 siRNA #2 were used.

**Supplementary Figure S2: Expression of BRCA1 upon BRCA1 silencing by Immunoflourescence.** a) Immunoflourescence of BRCA1 upon silencing BRCA1 in HCC1937. Two different siRNAs, BRCA1 siRNA #1 and BRCA1 siRNA #2 were used. Control siRNA served as control. Right panel represents the quantitation. b) Immunoflourescence of BRCA1 upon silencing BRCA1 in HCC1937/wt BRCA1. Two different siRNAs, BRCA1 siRNA #1 and BRCA1 siRNA #2 were used. Control siRNA served as control. Right panel represents the quantitation. c) Immunoblot analysis of BRCA1 in Control siRNA and BRCA1 siRNA #1 silenced HCC1937 and HCC1937/wt BRCA1 cells.

**Supplementary Figure S3: Expression of β-hCG upon BRCA1 silencing in different breast cancer cells by RT-qPCR.** RT-qPCR analysis of BRCA1, CGB5 and CGB7 expression in a, b) SUM149, c, d) MX1 and e, f) MCF-7 upon silencing BRCA1. Expression was normalized to control siRNA transfected MCF-7, MX1 and SUM149 respectively. Two different siRNAs, BRCA1 siRNA #1 and BRCA1 siRNA #2 were used.

**Supplementary Figure S4: Expression of BRCA1 upon BRCA1 silencing in MX1, MCF7 and SUM149 cells by Immunoflourescence.** a) Immunoflourescence analysis of BRCA1 in Control siRNA and BRCA1 siRNA #1 and #2 silenced MX1. b) Immunoflourescence analysis of BRCA1 in Control siRNA and BRCA1 siRNA #1 silenced MCF7. c) Immunoflourescence analysis of BRCA1 in Control siRNA and BRCA1 siRNA #1 silenced SUM149.

**Supplementary Figure S5: No mutation in BRCA1 exon 11 of BRCA1 floxed transgenic mice** a) BRCA1 floxed transgenic mice showing the orientation of LoxP sites. b) Sequencing of brca1 exon 11 in skin tumor samples of BRCA1 floxed male mouse.

**Supplementary Figure S6: Expression of EMT markers with respect to β-hCG in BRCA1 mutated cells.** a) Expression of CGB5 and CGB7 in HCC1937 and HCC1937/wt BRCA1 by RT-qPCR and ELISA after β-hCG silencing. Expression was normalized to scrambled siRNA. b) Immunoflourescence of Vimentin and E-cadherin in HCC1937, HCC1937/wt BRCA1 upon exogenous supplementation of β-hCG. Counter stain is done using DAPI. c) Expression of EMT markers in HCC1937 control vector transfected (HCC1937 C.vec), HCC1937 β-hCG transfected (HCC1937 β), HCC1937/wt BRCA1 control vector transfected (HCC1937/wt BRCA1 C.vec) and HCC1937/wt BRCA1 β-hCG transfected (HCC1937/wt BRCA1 β) cells by RT-qPCR. d) RT-qPCR analysis of EMT markers (Vimentin and E-cadherin) upon silencing the endogenous β-hCG expression. Csi represents Scrambled siRNA and βsi represents β-hCG siRNA. All the experiments were done in triplicates. All error bars in the graphs represent s.d.

**Supplementary Figure S7: β-hCG induces stemness in SUM149.** a) Sphere formation upon β-hCG over expression in SUM149 cells. b) Immunoflourescence analysis of OCT4, Slug and Vimentin in spheres derived from SUM149 β cells. c) RT-qPCR analysis of stem cell markers (Nanog, SOX2 and OCT4) in the spheres and adherent cells of β-hCG over expressed SUM149. d) RT-qPCR analysis of EMT markers (Zeb, Vimentin, Twist, Snail and fibronectin) in the spheres and adherent cells of β-hCG over expressed SUM149.

**Supplementary Figure S8: β-hCG induces signaling irrespective of LHCGR.** Analysis of β-hCG, LHCGR in a cohort of breast cancer samples from TCGA dataset. Expression of LHCGR in breast cancer cell lines by b) RT-PCR and c) Immunoflourescence. d) Ability of cells to attach the adherent plates and attain the morphology in serum free media, in the presence of full hCG (α+β) and in the presence of β-hCG. e) IHC analysis of β-hCG, LHCGR in the mouse mammary tumor of BRCA1 floxed mouse skin tumor tissue

**Supplementary Figure S9: Expression of β-hCG in BRCA1 related cancers.** a) Expression of β-hCG (CGB5) in different cancer types was analyzed using cBioPortal. Altertions in CGB5 with minimum 1% in different cancers was represented in the graph. b) Expression of β-hCG (CGB5) in breast cancer sub types was analyzed using oncomine.
