# Supplementary figures and images for: BRCA1 regulation on β-hCG: a mechanism for tumorigenicity in BRCA1 defective breast cancer
Source: Oncogenesis. 2017 Sep 4;6(9):e376–. doi: 10.1038/oncsis.2017.75 (PMC5623901; doi:10.1038/oncsis.2017.75)

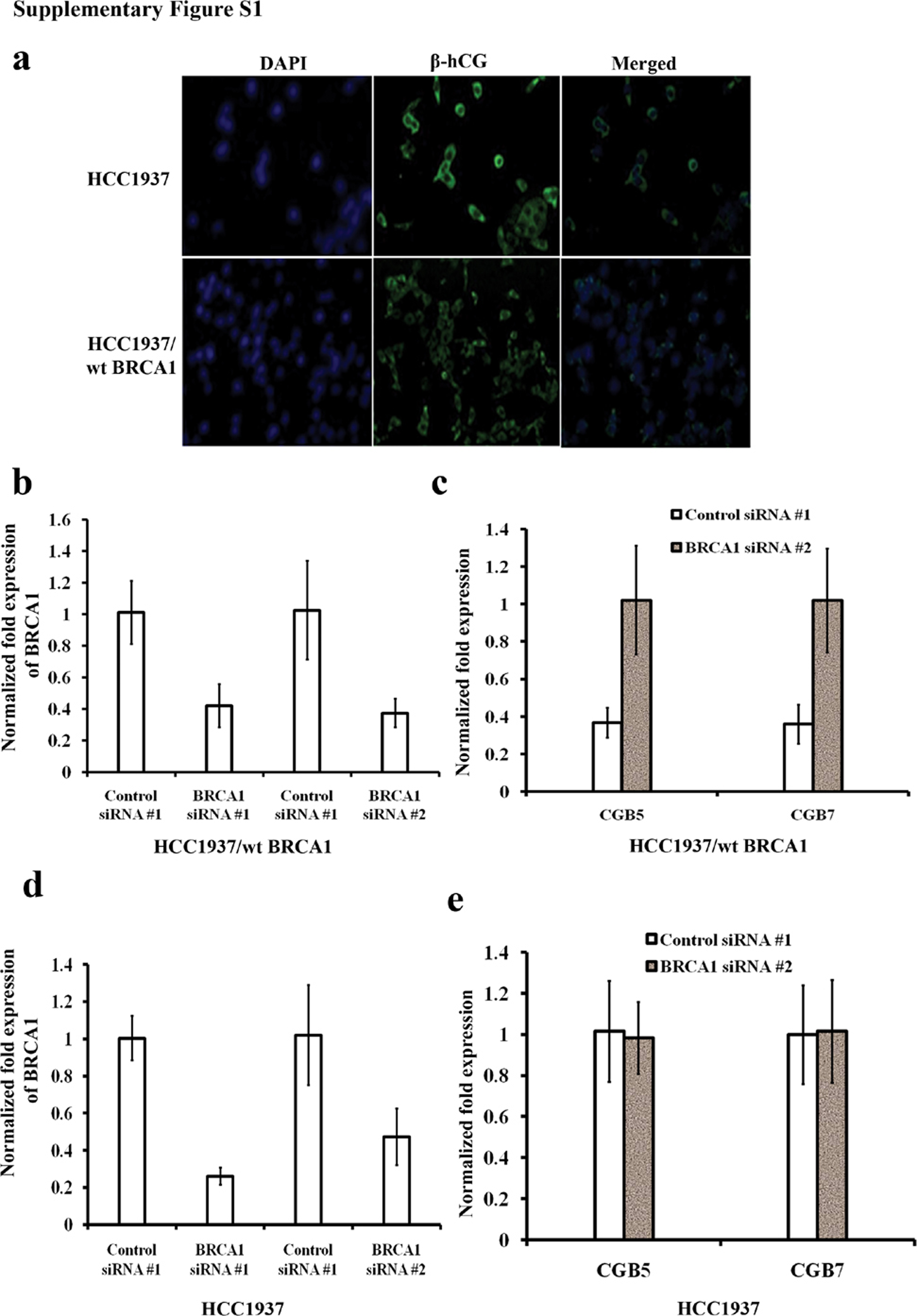

Supplement: Supplementary Figure 1 [file oncsis201775x2.tif]

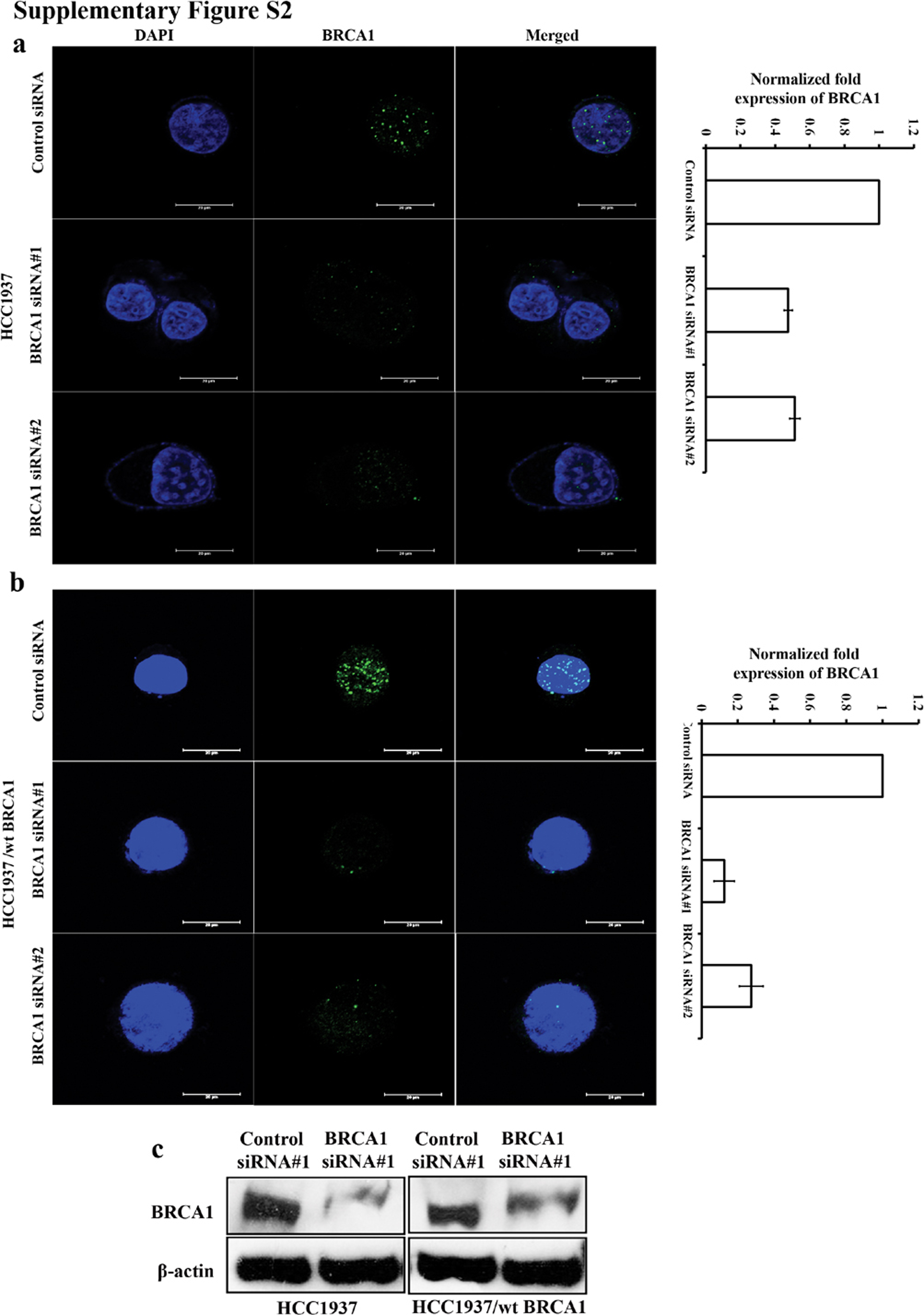

Supplement: Supplementary Figure 2 [file oncsis201775x3.tif]

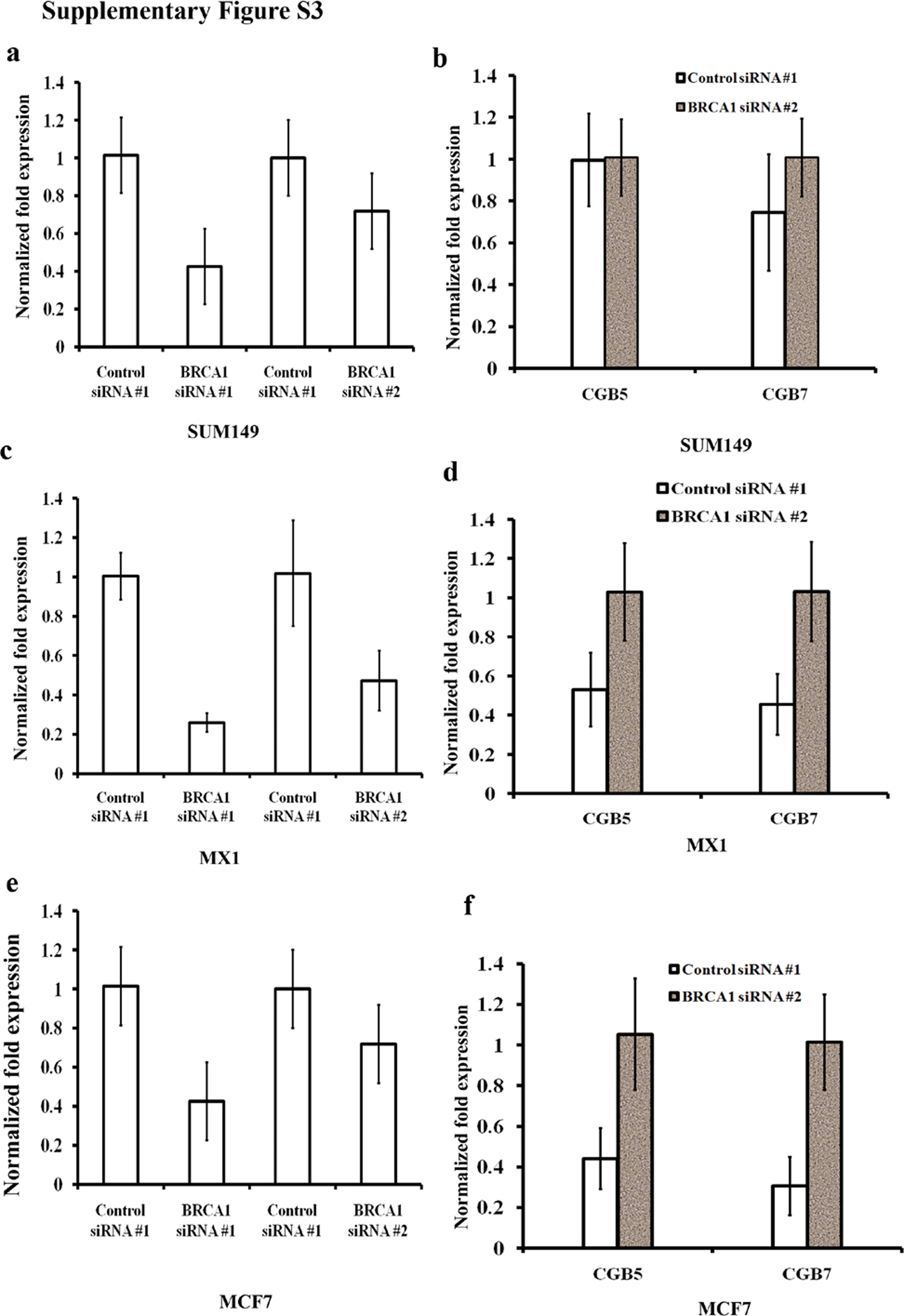

Supplement: Supplementary Figure 3 [file oncsis201775x4.tif]

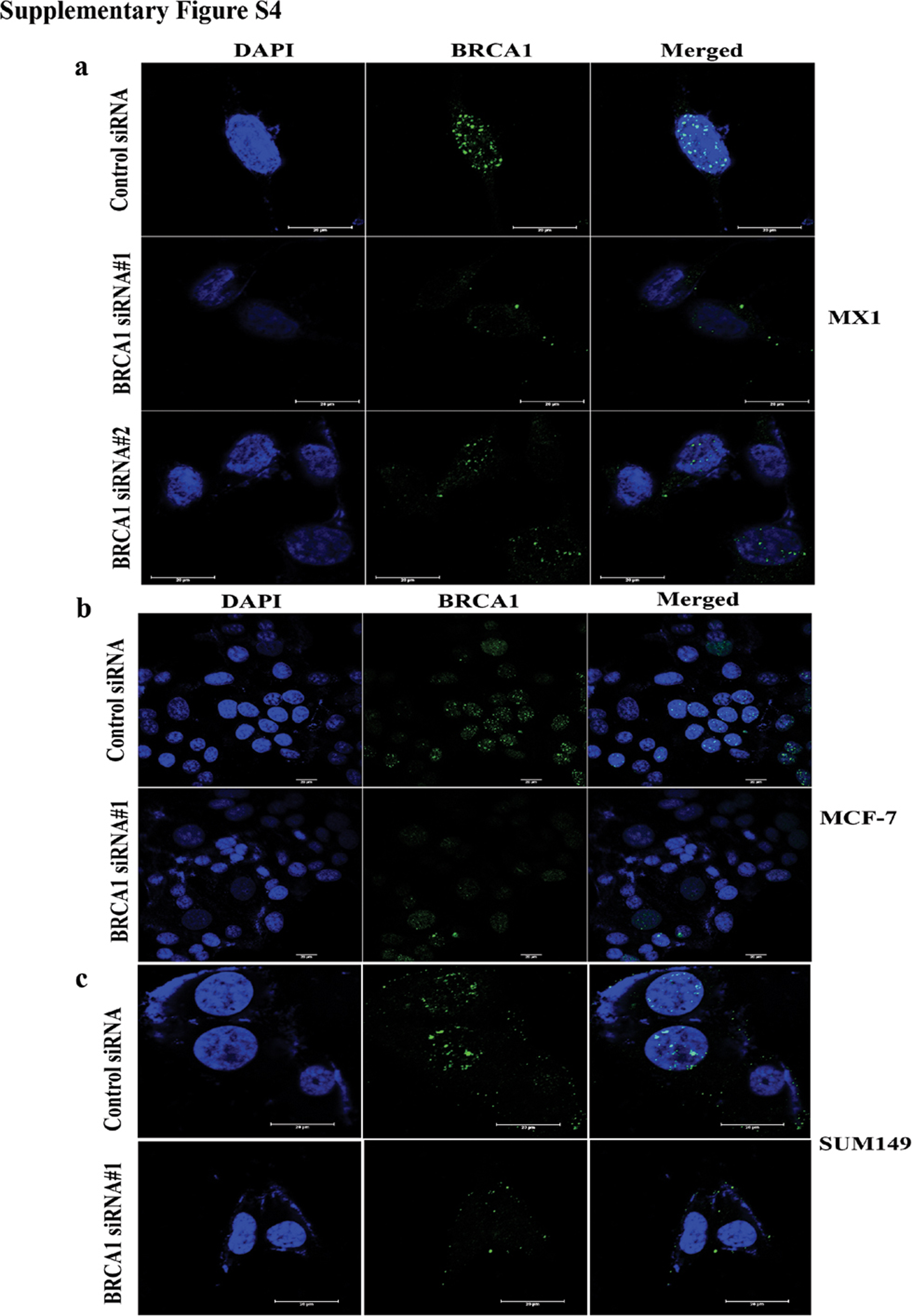

Supplement: Supplementary Figure 4 [file oncsis201775x5.tif]

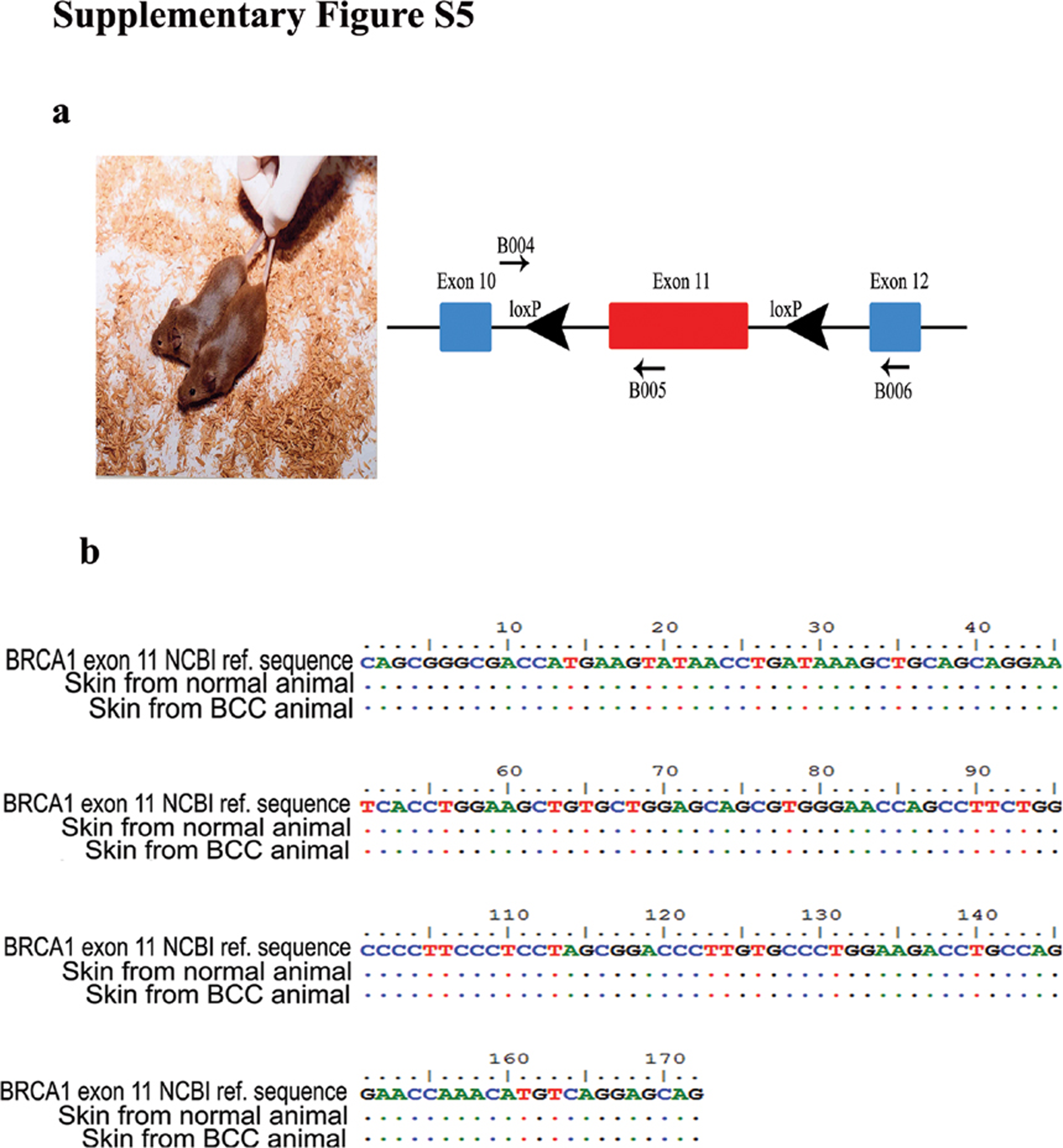

Supplement: Supplementary Figure 5 [file oncsis201775x6.tif]

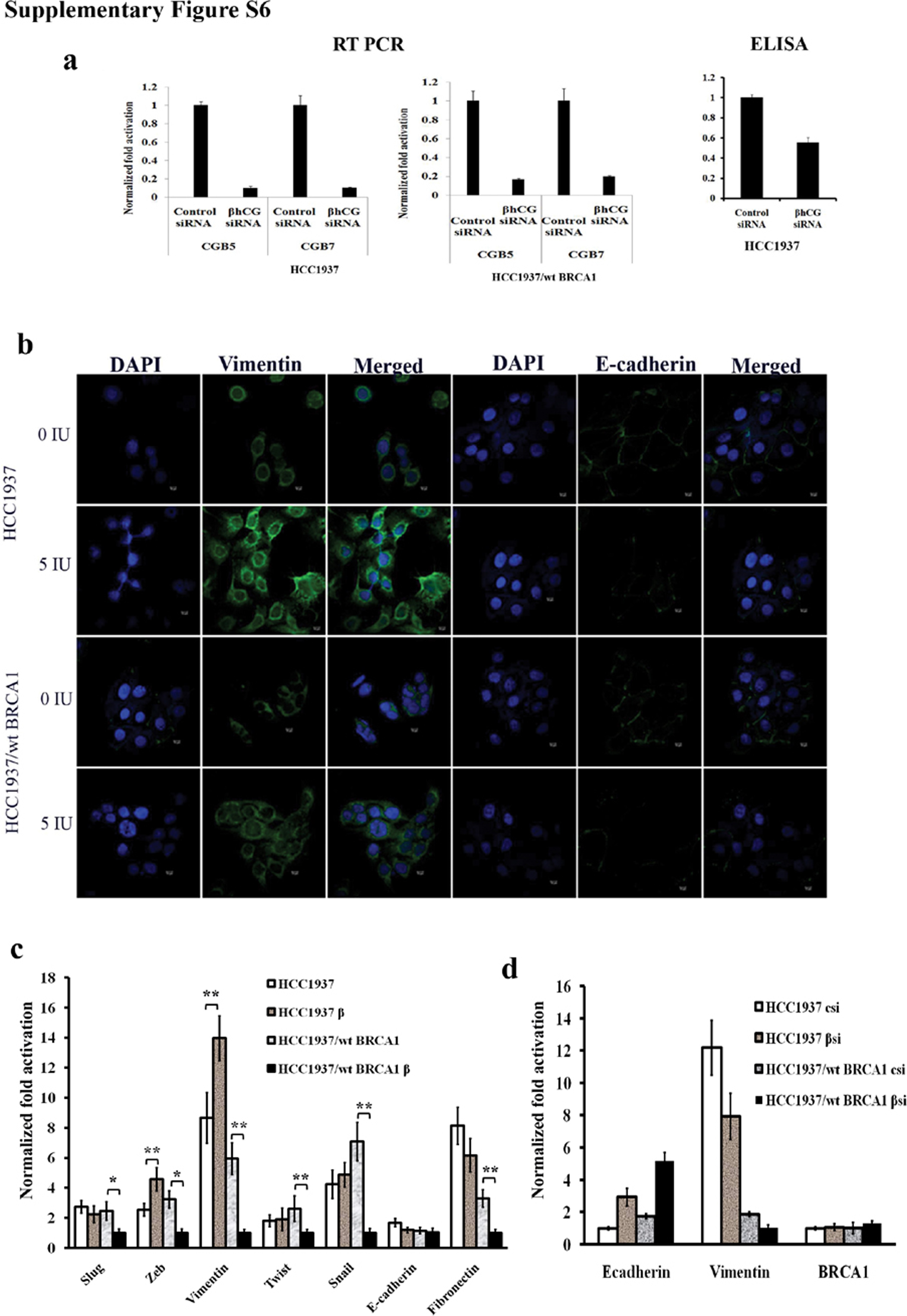

Supplement: Supplementary Figure 6 [file oncsis201775x7.tif]

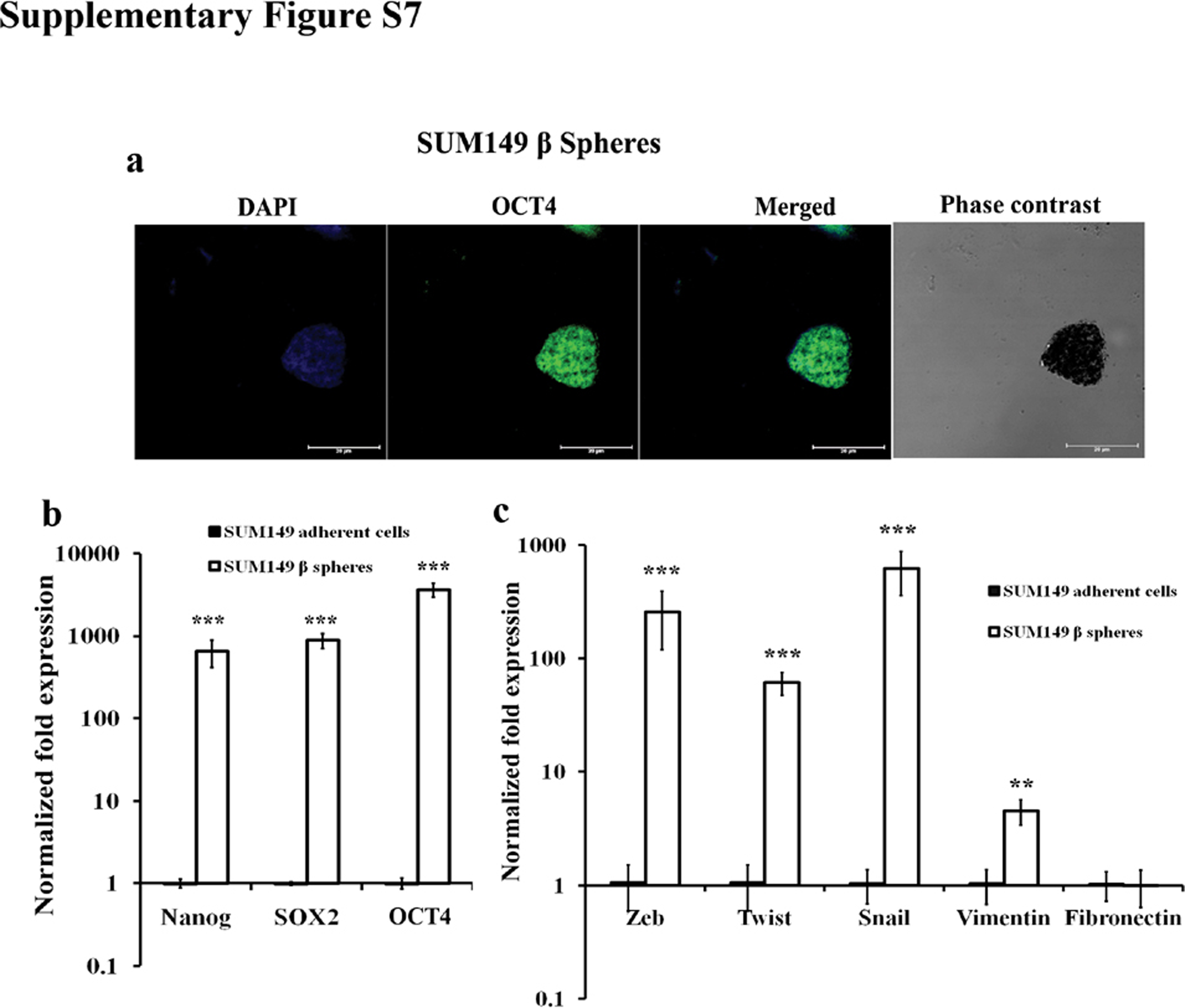

Supplement: Supplementary Figure 7 [file oncsis201775x8.tif]

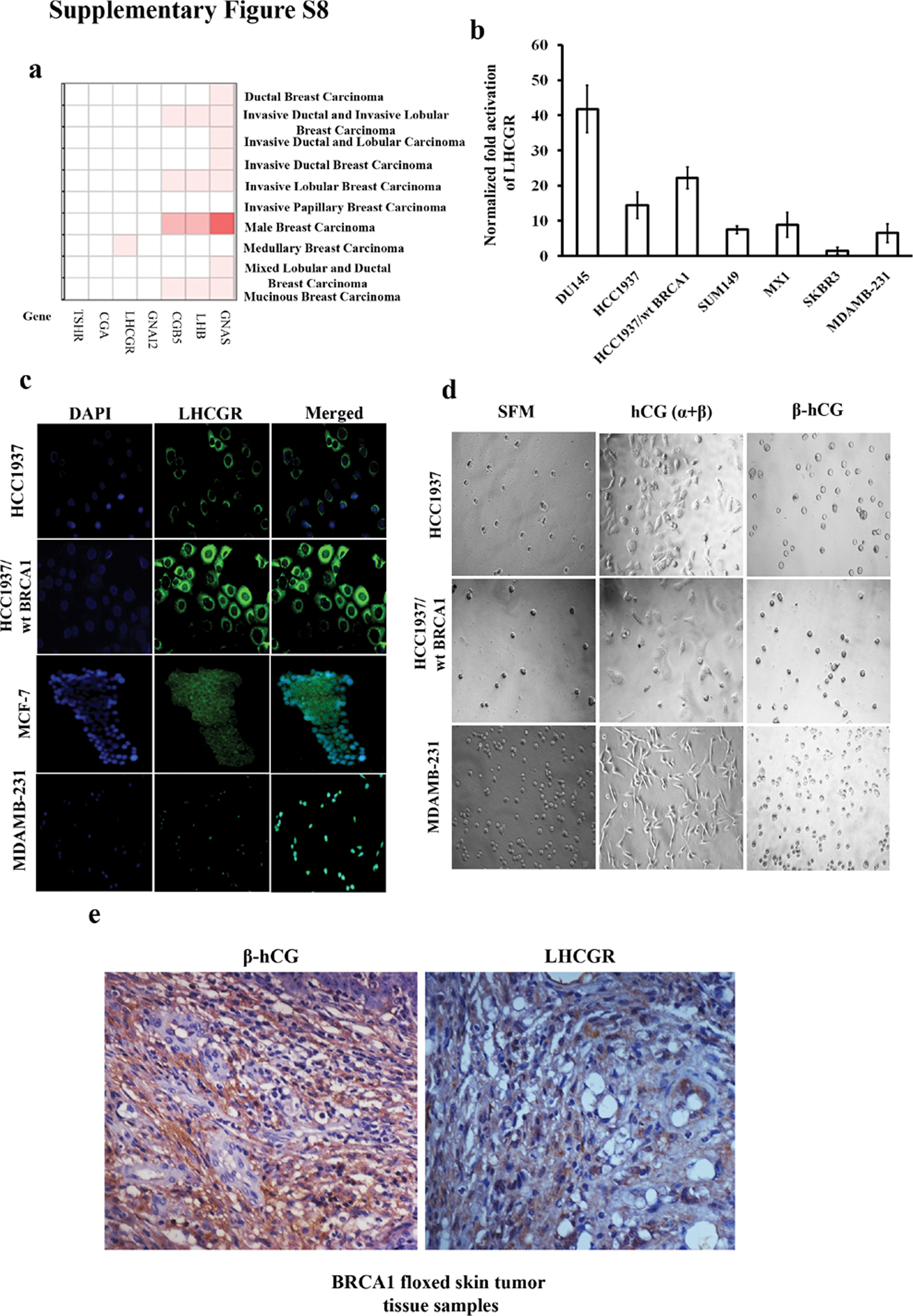

Supplement: Supplementary Figure 8 [file oncsis201775x9.tif]

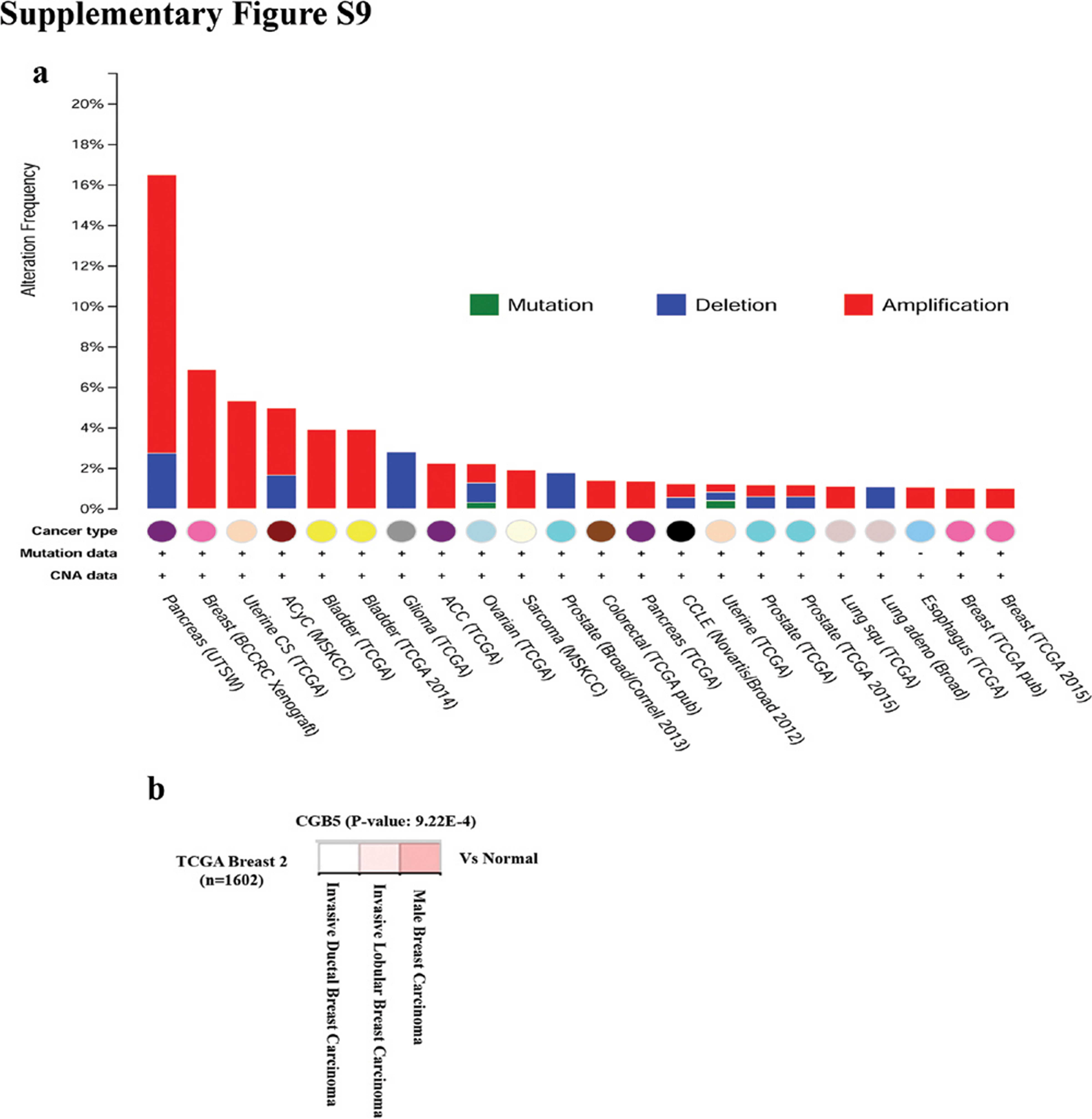

Supplement: Supplementary Figure 9 [file oncsis201775x10.tif]
